# 782 The Effect of Medical Comorbidites on Mortality in Burn Patients

**DOI:** 10.1093/jbcr/irad045.257

**Published:** 2023-08-29

**Authors:** Shevonne S Satahoo, Barbara Okeke, Joyce I Kaufman, Geraldine Nabeta, Louis R Pizano, Carl I Schulman

**Affiliations:** University of Miami, Miami, Florida; St. George’s University, Miami, Florida; University of Miami, Miami, Florida; St. George’s University, Miami, Florida; University of Miami, Miami, Florida; University of Miami-Jackson Memorial Hospital, Miami, Florida

## Abstract

**Introduction:**

Age, percent total burn surface area (TBSA) burn, and presence of inhalation injury have been well accepted as prognostic factors associated with burn injury. However, there may be additional comorbidities that affect mortality to consider. Recently published data include comorbidities specifically in the elderly population or focus on a specific comorbidity. As such, this analysis sought to identify comorbidities with a more comprehensive approach while focusing on the adult population.

**Methods:**

The National Inpatient Sample was queried for all patients with age 18-65 years with ICD-9 codes for total body surface area (TBSA) burn ≥ 20% and non-elective admissions. Years included were 2013- third quarter of 2015. Age, race, comorbidities and mortality were recorded. Cases with missing data for mortality were excluded. Statistical analysis was done with Chi-Square testing. Mortality was then used to perform a binary logistic regression using these variables.

**Results:**

There were 5545 weighted cases. The median age was 41.0 years (IQR 29-52 years). Mortality was 14.7%. The presence of AIDS, chronic blood loss anemia, chronic pulmonary disease, congestive heart failure, peptic ulcer disease (excluding bleeding), peripheral vascular disease, renal failure, rheumatoid arthritis/collagen vascular disease, and solid tumor without metastasis were not found to be statistically significant. The significant variables were then entered into a binary regression model on mortality. For the regression analysis, age, gender and race were also included. Age and race were statistically significant (p < 0.001 for both), while gender was not (p=0.072). While significant on univariate analysis, alcohol abuse (p=0.059), diabetes with chronic complications (p=0.244), metastatic cancer (p=0.999), psychoses (p=0.053) and valvular disease (p=0.998) were no longer significant on multivariate analysis. The results of the significant comorbidities are listed in the table. In those who died, there were increased odds of having coagulopathy, uncomplicated diabetes, liver disease, paralysis, pulmonary circulation disorders and obesity.

**Conclusions:**

Identification of these significant comorbidities potentially highlights the need for updated models to assist clinicians in determining risk of mortality in burn patients. Theses comorbidities also point to the importance of resources for primary care physicians to optimize these patient populations and potentially minimize the risk of complications should patients become injured.

**Applicability of Research to Practice:**

